# Temporal topic model for clinical pathway mining from electronic medical records

**DOI:** 10.1186/s12911-024-02418-1

**Published:** 2024-01-23

**Authors:** Wei Li, Xin Min, Panpan Ye, Weidong Xie, Dazhe Zhao

**Affiliations:** 1 School of Computer Science and Engineering, Northeastern University, Shenyang, 110000 China; 2 Key Laboratory of Intelligent Computing in Medical Image (MIIC), Northeastern University, Shenyang, 110000 China

**Keywords:** Clinical pathway mining, Topic models, Latent Dirichlet Allocation, Temporality

## Abstract

**Background:**

In recent years, the discovery of clinical pathways (CPs) from electronic medical records (EMRs) data has received increasing attention because it can directly support clinical doctors with explicit treatment knowledge, which is one of the key challenges in the development of intelligent healthcare services. However, the existing work has focused on topic probabilistic models, which usually produce treatment patterns with similar treatment activities, and such discovered treatment patterns do not take into account the temporal process of patient treatment which does not meet the needs of practical medical applications.

**Methods:**

Based on the assumption that CPs can be derived from the data of EMRs which usually record the treatment process of patients, this paper proposes a new CPs mining method from EMRs, an extended form of the traditional topic model - the temporal topic model (TTM). The method can capture the treatment topics and the corresponding treatment timestamps for each treatment day.

**Results:**

Experimental research conducted on a real-world dataset of patients’ hospitalization processes, and the achieved results demonstrate the applicability and usefulness of the proposed methodology for CPs mining. Compared to existing benchmarks, our model shows significant improvement and robustness.

**Conclusion:**

Our TTM provides a more competitive way to mine potential CPs considering the temporal features of the EMR data, providing a very prospective tool to support clinical diagnostic decisions.

**Supplementary Information:**

The online version contains supplementary material available at 10.1186/s12911-024-02418-1.

## Background

### Introduction

Clinical pathways (CPs) refer to the treatment pattern that the medical staff in a hospital must follow for a disease, so that patients receive medical services such as examination, surgery, treatment and nursing according to the pattern from admission to discharge, and thereby achieve the purposes of saving medical resources and improving medical efficiency. The earlier CPs were constructed manually relying mainly on the clinical knowledge of experts. The designed CPs have some disadvantages such as static and non-adaptive, which make them difficult to perform in clinical treatment [1]. In recent years, with the availability of EMRs, experts are taking great interest in leveraging personalized medical data to mine CPs. Therefore, the mining of CPs has shifted from knowledge-driven to data-driven.

**Fig. 1:**
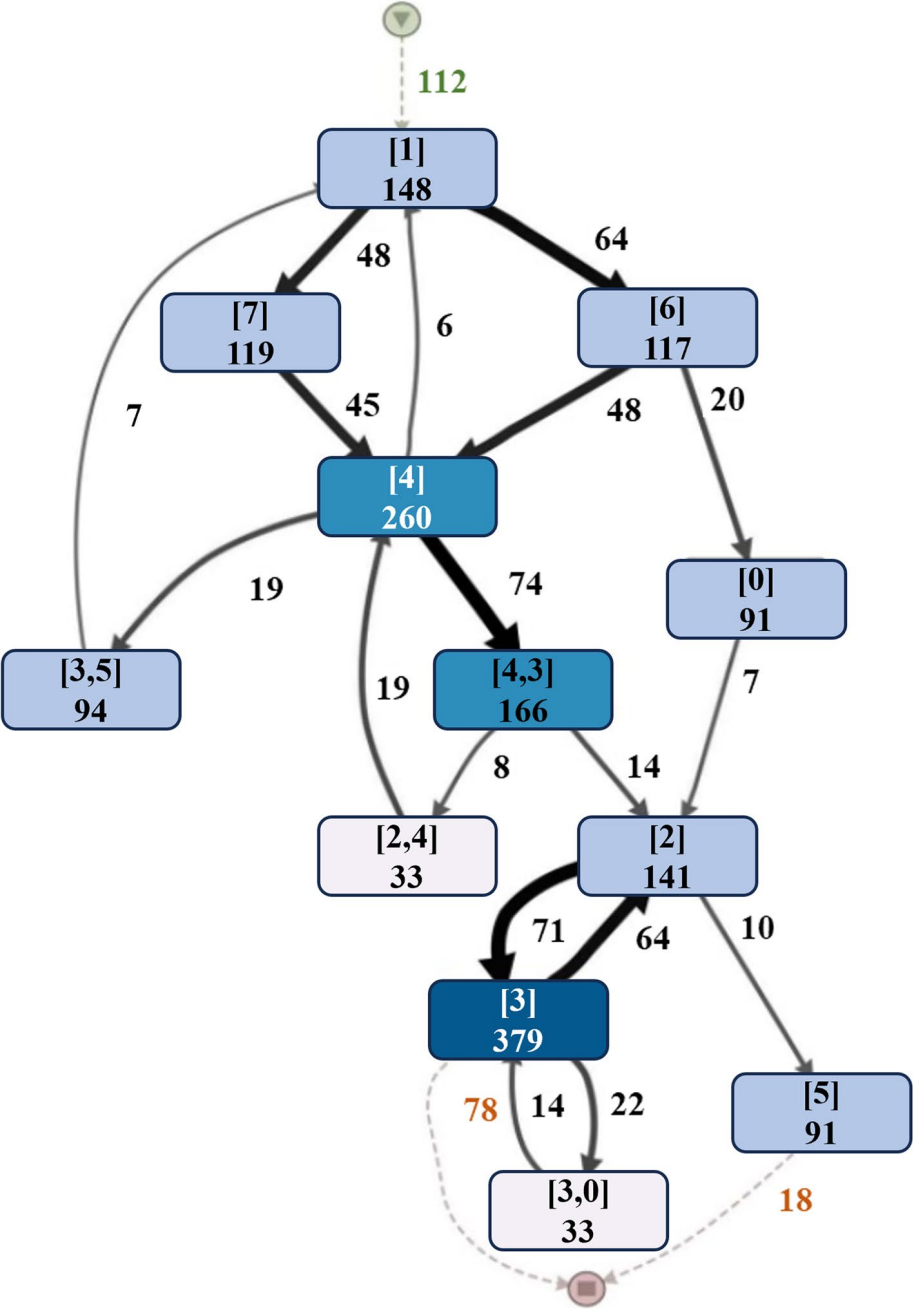
An illustration of CP about intracerebral hemorrhage (ICH) in research [6]

Compared to doctor-designed CPs, the mining of CPs from EMRs data represents objective information and knowledge that helps design more adaptive CPs. The latent CPs in EMRs represents patients receiving treatment according to a certain pattern, which are similar to the explicit treatment knowledge that cannot be extracted using existing methods. Due to the limitations of data and technology, data-driven CPs mining is still in the exploration stage. In terms of data, the private nature of medical data makes it difficult to obtain reliable and high-quality data. Moreover, there is some variability in the format of EMRs data from different periods due to different recording habits of medical staff, which makes it difficult to directly be used for the CPs mining. In terms of technology, most of the existing researches focus on analyzing clinical data with process mining techniques [2–4], which has been extensively studied in the field of business process management and which tries to extract important and useful information from EMRs data. In clinical practices, many hospitals’ EMRs systems record patients’ treatment processes that perform doctors-assigned CPs, whereby each treatment process corresponds to a specific disease. However, the mining of CPs is in general a challenging task [5], as the diversity and complexity of treatment behaviors in treatment processes is much higher than that in ordinary business processes. Therefore, the mining of CPs with processes mining often produces spaghetti-like patterns, which are difficult to understand by clinical experts and are not suitable for the analysis of CPs or directly to assist doctors in their diagnosis. As shown in Fig. 1, node number and link number represent the treatment topic code and the patient number, respectively. The mining of CPs with processes mining generates multiple circular paths, which are difficult to interpret in the clinical process.

In order to solve the above problems, more and more researchers try to adopt topic models for CPs mining. Huang et al. have done a lot of research work [7–10] on CPs mining based on topic models. However, these CPs mining methods based on topic models focus on the discovery of treatment patterns without consideration of temporality, making it difficult to meet the requirements of CPs on temporal relationships. Moreover, Xu et al. [6, 11] have tried to combine process mining with topic models to first identify the treatment topics of each treatment day in the treatment process, and then discover the temporal relationships in treatment topics. As shown in Fig. 1, although these methods can better compensate for the lack of topic models as compared to traditional topic models [12], CPs generated by process mining are still difficult to be understood by clinical doctors because of the complexity of their processes.

To this end, we propose a novel temporal topic model (TTM) for the clinical pathway mining from EMRs. Firstly, we consider the patients’ treatment process for a disease in EMRs as an ensemble consisting of many treatment days. Secondly, our model generates a treatment topic from a multinomial distribution conditioned on treatment day. Finally, our model generates the corresponding treatment timestamps and treatment activities from other multiple distributions based on latent treatment topics. In this complete probabilistic generative model, the model is able to distinguish treatment topics and their treatment timestamps for different treatment days with the same treatment activity, and discovers latent CPs consisting of three tuples, where the three tuples include treatment topics, treatment timestamps and the probability distribution.

In summary, the main points of the paper are:

- We propose an extended form of traditional LDA, i.e., temporal topic model to capture the temporal relationships in CPs mining.
- Our proposed model organizes the treatment days into a number of treatment topics over the treatment process, increases data granularity, and combines corresponding treatment timestamps to form simple, interpretable, and temporal CPs.

The rest of the paper is organized as follows: “Method” section presents the related work. “Results” section describes our proposed methodology. “Discussion” section performs the experimental evaluation and analysis. “Conclusion” section discusses the contribution, novelty and limitations of the proposed method. Finally, “Declarations” section concludes the entire paper.

### Related work

In this section, we summarize the related work into two categories, process mining and topic models, and we highlight what makes our work different from previous work.

#### Process mining

The most relevant direction for our research work is healthcare business process mining [13, 14]. As a general method of business process analysis [3, 15], the main idea of process mining is to mine the process knowledge of business activities from business execution logs. For instance, the identification of frequent treatment patterns from hospital care logs can be used to analyze and improve CP implementation.

Process mining has received increasing attention from the researchers because it plays an important role in the analysis of CPs and other types of healthcare processes. In [2], Yang et al. proposed a process mining algorithm to contribute to the automated and systematic detection of healthcare fraud and abuse of CP. In [16], Mans et al. applied process mining to discover the treatment patterns of stroke patients in different hospitals. In [17], Huang et al. developed a new process mining algorithm to derive brief summaries from clinical event logs.

Due to the greater diversity of medical behaviours in clinical processes than in ordinary business processes, the adoption of traditional process mining techniques can produce spaghetti-like process patterns which are difficult to interpret by clinical experts [6, 11]. These process models lack a certain applicability to support clinical process analysis and improvement in actual practice. Compared to existing process mining, we adopt an improved topic model to mine clinical pathways, which avoids generating complex process patterns.

#### Topic models

Automated discovery of executive treatment patterns based on massive, unique clinical data is attracting more and more research for its value in clinical pathway design [1, 18, 19]. To tackle the high-dimensional, sparse, and noisy characteristics of medical data, some researchers have employed topic models to perform representation learning on medical data, and then identify the core treatment patterns based on the representation learning. Topic models have been applied to unsupervised text representation learning for non-connected documents firstly [12], which considers a document consists of different words with several topic. Such a probabilistic model is suitable for extracting the hidden topic semantics from medical data. Chen et al. [20] proposed LDA to perform topic mining from hospital charge item data, and it can significantly distinguish the similarities and differences between these topics by comparing and analyzing the data from different hospital informatics. Huang et al. conducted a lot research work on clinical treatment pattern mining based on topic models [7–9]. The research [7] regarded each hospitalisation as a document and each activity as a word, and the latent treatment patterns were mined by the topic model. In the study [9], the research team put the examination results of treatment activities into a topic model, making the discovered treatment patterns contain richer information.

Recent researches have attempted to add temporal information to the topic model and develop a variant of the topic model, the temporal topic model. In the study [8], the temporal information of treatment activities was imported into the topic model, and this variant of the topic model was leveraged to mine treatment patterns with certain temporal sequences from the data. In [6, 11], Xu et al. attempted to combine process mining and topic models, which first identified the treatment topics of each treatment day in the treatment process through topic models, and then leveraged process mining to discover the temporal relationships between these topics to achieve the CPs mining.

However, these CPs mining methods based on topic models focus on the discovery of treatment patterns, i.e. the discovery of treatment patterns containing specific treatment activities, and the treatment patterns lack consideration of temporality to meet the needs of CPs for temporal relationships. Even though some methods attempt to combine topic models and process mining, which can capture certain temporal relationships, the resulting process models are still difficult to understand by clinical experts and implement in concrete practice. Compared to existing topic model mining methods, our method considers temporal information in the patient’s treatment process and mines clinical pathways which are easier to interpret and implement.

## Method

In this section, we first introduce the notation and definitions, and describe the CPs mining problem. Then, we briefly introduce the traditional topic model. Finally, we describe our model TTM in detail.

### Notations and definition

The purpose of this study was to mine latent CPs from EMRs in hospitals. In particular, we assume that clinical activities are recorded by treatment day timestamp order in EMRs so that each treatment day contains specific treatment activities. Before defining some concepts, Table 1 summarizes some important mathematical notations. Some concepts in the clinical process are formulated as follows.

#### Definition 1

(Treatment Activities) Let $\mathcal {A}$ denotes the set of all treatment activities for a kind treatment option of specific diseases. We define *a* to denote the treatment activities in the treatment process, i.e., $a \in \mathcal {A}$.

#### Definition 2

(Treatment Days) Let $\mathcal {D}$ denotes the set of treatment days in the treatment process. We define *d* to denote the treatment day in the treatment process, i.e., $d \in \mathcal {D}$. The treatment days are the non-empty sets of clinical activities performed on a particular patient, i.e., $d=\left[ a_{1}, a_{2}, \ldots , a_{|d|}\right]$, where $a_{i} \in \mathcal {A}(1 \le i \le |d|)$ denotes the particular clinical activity.

#### Definition 3

(Treatment processes) Let $\mathcal {L}$ denotes the whole treatment processes for a kind of disease in the dataset. We define $\sigma$ to denote a specific treatment process for a specific patient, i.e., $\sigma \in \mathcal {L}$. The treatment processes are non-empty sets of treatment days performed on a particular patient, i.e., $\sigma =\left[ d_{1}, d_{2}, \ldots , d_{|\sigma |}\right]$, where $d_{i} \in \mathcal {D}$ denotes a particular treatment day.

#### Definition 4

(Treatment timestamp) Let $\mathcal {T}$ denotes the set of treatment timestamps and *t* denotes each treatment timestamp. Each treatment day has an occurring timestamp, it corresponds to the treatment topic.

**Table 1:** Mathematical notations

Symbol	Description
$\mathcal {A}$	The set of all treatment activities;
*a*	The treatment activity;
$\mathcal {D}$	The set of all treatment days;
*d*	The treatment day;
*K*	The number of treatment topics;
$N_{d}$	The number of all treatment activities in a treatment day;
$\theta$	The probability distribution of topic in treatment day;
$\varphi$	The probability distribution of timestamp in treatment topic;
$\phi$	The probability distribution of activity in treatment topic;
$\alpha , \delta , \beta$	The hyper-parameters;
*z*	The treatment topic;
*t*	The treatment timestamp of treatment day;
*T*	The universe of treatment days;
$\mathcal {L}$	The whole treatment processes;
$Z_{N}$	The normalization factor;
*N*	The top activity number in the ranking results;
$n_{d,k}$	The number of times that the day *d* is assigned to topic *k*;
$q_{k,t}$	The number of times that the timestamp *t* is assigned to topic *k*;
$m_{k,t,a}$	The number of times that the activity *a* is assigned to topic *k* with timestamp *t*;
$\sigma$	A treatment process for a specific patient;
$\mathcal {T}$	The set of treatment timestamp;
*C*	The triples form of discovered CP;

**Fig. 2:**
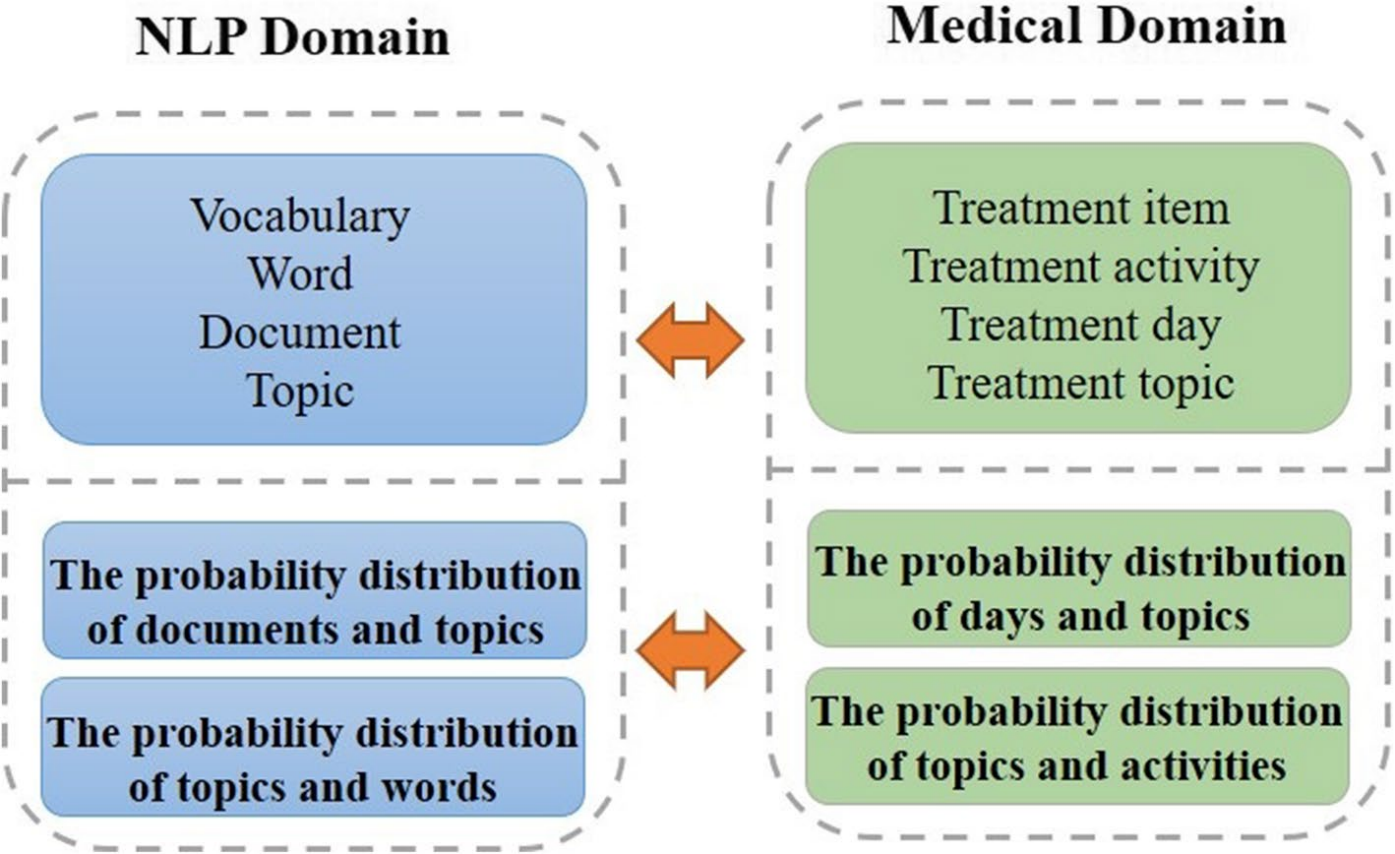
The mapping relationship of topic models in natural language processing (NLP) domain and medical domain

### Problem formulation

As shown in Fig. 2, we extend the topic model from natural language processing (NLP) to the medical domain, trying to leverage the topic model to mine CPs. The purpose of this study is to first generate topics for each treatment day based on the patients’ treatment processes, then generate the corresponding timestamps and contained activities based on the topics, and finally form multiple three tuples of topics and timestamps into a CP for a specific disease. In particular, the process of generating CPs starts with the topic *z* selected from the distribution $\theta$ for the given day *d*. Given a probability distribution $\varphi$ of timestamp *t* occurring in topic *z*, the corresponding timestamp is generated by sampling the topic from the distribution. Similarly, given a probability distribution $\phi$ of activity *a* occurring in topic *z*, activity is generated by sampling topics from that distribution.

The generated CPs consists of various triples of $|T|$ treatment days, $\varvec{C}=\left\{ \left[ \left( z_{1}, t_{1}, p_{1}\right) , \cdots ,\left( z_{k}, t_{1}, p_{k}\right) \right] , \ldots ,\left[ \left( z_{1}, t_{|T|}, p_{1}\right) , \cdots ,\left( z_{k}, t_{|T|}, p_{k}\right) \right] \right\}$, where $z_{i}=\left( a_{1}, a_{2}, \ldots , a_{|d|}\right)$ denotes the distribution of treatment activities for each treatment topic, $t_{i}$ denotes the treatment timestamp, and $p_{i}$ is determined by the treatment day-topic probability distribution $\theta$.

**Fig. 3:**
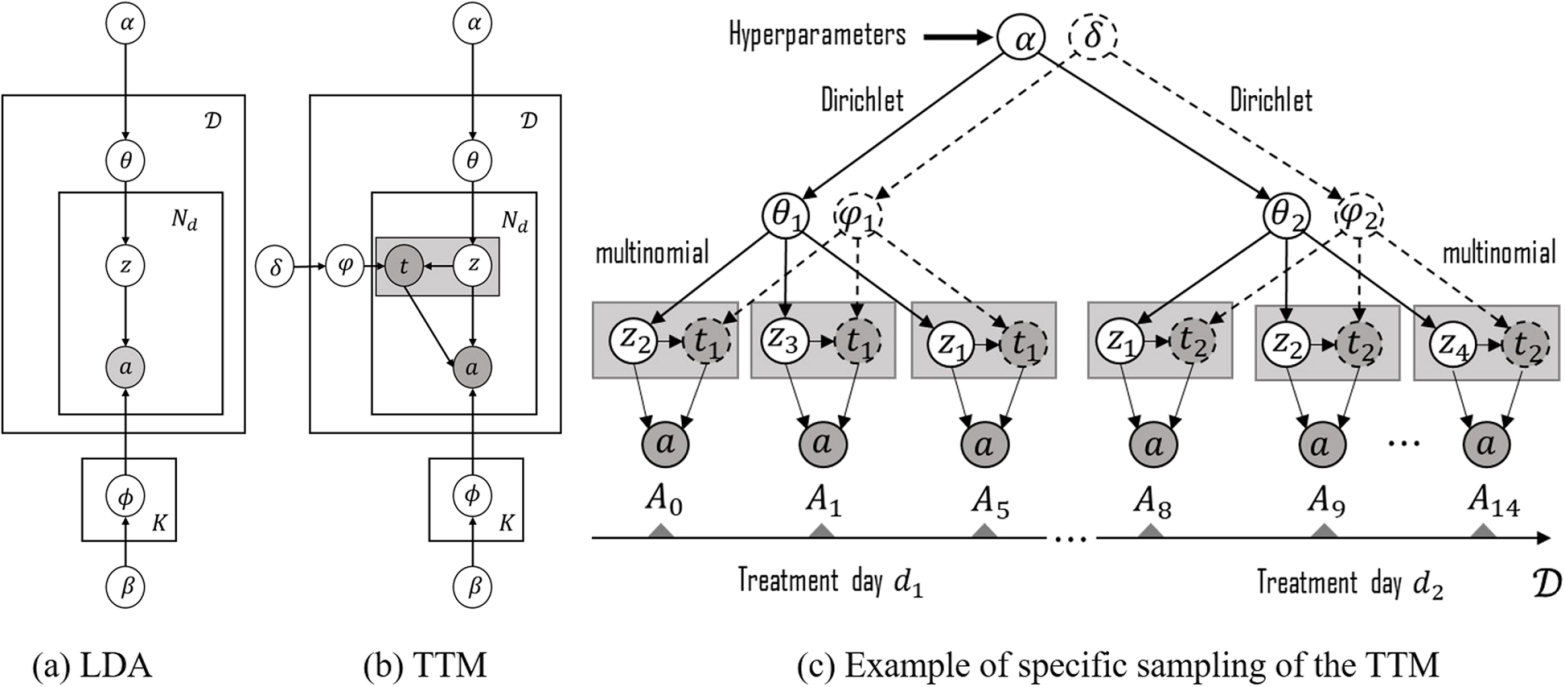
Graphical representation of two probabilistic models (**a**) traditional LDA, (**b**) TTM, and (**c**) an example of the generative process with TTM

### Latent Dirichlet Allocation

Topic models are a powerful tool in natural language processing, originally developed to represent text documents. The Latent Dirichlet Allocation (LDA) is a probability topic model based on the dirichlet distribution [12]. The LDA topic model presents each document as a multinomial distribution of topics, and each topic is presented as a multinomial distribution of words. It is a probabilistic generative model, which is a kind of unsupervised learning.

In the previous research work, Xu et al. [6] proposed a generated statistical model, the Topic-Based Clinical Pathway Mining Model (TCPM), which models the treatment day of a patient’s treatment process by *K* latent treatment topics and finally combines with process mining to achieve the CPs mining. As shown in Fig. 3(a), where $\theta$ and $\phi$ denote the probability distributions of topics in treatment days, and the probability distributions of treatment activities in topic, respectively. The hyper-parameters are denoted by $\alpha$ and $\beta$, respectively. In particular, the $\alpha$ is the Dirichlet prior of the probability distribution $\alpha$, which can be interpreted as the prior observation counts for the number of times the topic was sampled from the patient’s treatment day before any treatment activity was observed. The $\beta$ is the Dirichlet prior for the probability distribution $\phi$, which can be interpreted as the prior observation counts for the number of times a particular treatment activities were sampled from the treatment subject before any the actual treatment activities were observed.

Although the TCPM leverages the treatment days in a patient’s treatment process, it fails to take full advantage of the temporal relationships in the different treatment days and the generated topics are disordered. The detailed process is as follows:
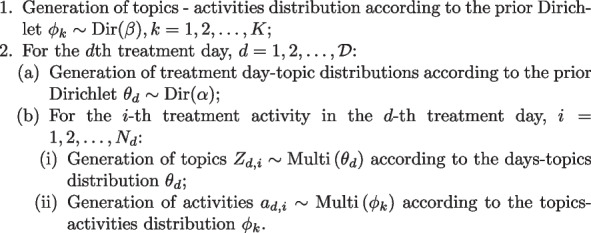


According to the previous work [21, 22], following the initialization of the hyper-parameters, Gibbs sampling is generally applied to iteratively draw samples from the probability distribution of each treatment topic $z_{d, i}$:

1 $$\begin{aligned} P\left( z_{d, i}=k \mid \textbf{z}_{-i}, a\right) \propto \frac{n_{d, k}^{-i}+\alpha _{k}}{\sum _{k \in K} n_{d, k}^{-i}+K \alpha } \times \frac{m_{k, a}^{-i}+\beta _{a}}{\sum _{a \in A} m_{k, a}^{-i}+|A|\beta } \end{aligned}$$

where $z_{d, i}=k$ denotes the assignment of the *i*th treatment activity of treatment day *d* to treatment topic *k* during patient treatment, and $\textbf{z}_{-i}$ denotes all treatment topics that do not contain the topic of the *i*th treatment activity. Furthermore, $n_{d, k}^{-i}$ denotes the number of treatment topics that occurred on treatment day *d* and did not contain the topic of the *i*th treatment activity, and $m_{k, a}^{-i}$ denotes the number of treatment activities assigned to topic *k* and did not contain the *i*th treatment activity.

After completing the Gibbs sampling, the two probability distributions $\theta _{d}$ and $\phi _{k}$ are calculated as follows.

2 $$\begin{aligned} \theta _{d}=\frac{n_{d, k}^{-i}+\alpha _{k}}{\sum _{k \in K} n_{d, k}^{-i}+K \alpha } \end{aligned}$$

3 $$\begin{aligned} \phi _{k}=\frac{m_{k, a}^{-i}+\beta _{a}}{\sum _{a \in A} m_{k, a}^{-i}+|A|\beta } \end{aligned}$$

The algorithm first assigns a random topic to each activity, updates the topic of each activity with Gibbs sampling, and then repeats the Gibbs sampling process to update the topics assigned for the iteration.

Our work can be seen as building on the previous earlier work in topic clinical pathway mining (TCPM). We will describe our work in detail in the next subsection. As an extended form of topic models, our proposed model is able to associate the treatment topics of each treatment day with the corresponding treatment timestamps and infer the impact of specific timestamps on clinical pathway mining.

### Temporal topic model

The TCPM method can capture the treatment topics for each treatment day in the patient’s treatment process. However, TCPM neither identifies the temporal nature of the treatment process nor the association between timestamp and topic. To this end, we propose an extended form of the LDA, the TTM, which models the contribution of treatment activity as well as treatment timestamps.

As shown in Fig. 3(b), the proposed temporal topic model can discover latent CPs, which are identified by the discovered treatment topics and their corresponding timestamps. The generation process of the model is similar to the standard LDA, which first generates the treatment activities to be performed, and then generates the corresponding treatment topics and the corresponding timestamps. For EMRs data recording the execution process of CPs, the treatment topic probability $\theta _{d}$ is derived for each treatment day according to the Dirichlet distribution, and each treatment topic distribution is associated with a multinomial distribution $\varphi _{k, d}$ on the treatment timestamps and a multinomial distribution $\phi _{k, d, a}$ on the treatment activities. Furthermore, the probability distributions $\theta _{d}, \varphi _{k, d}$ and $\phi _{k, d, a}$ correspond to the prior Dirichlet hyper-parameters $\alpha , \delta$, and $\beta$, respectively.

Figure 3(c) shows a possible generative process for treatment topics and corresponding treatment timestamps when modeled as TTM, which can be viewed as the expanded graphical model of the plate representation in (b), where the model can associate treatment timestamps and treatment themes and infer the contribution of timestamps to the discovery of treatment activities. The shaded and unshaded nodes here indicate the observed and latent variables, respectively. In this example, we assume that there are four latent treatment topics $z_{1}, z_{2}, z_{3}$ and $z_{4}$. A treatment process consists of a set of treatment days, which are spread along the time-line of length of stay, is mixed with four treatment topics.

Since it is very difficult to implement the exact derivation of the topic model, we use an approximate derivation based on Gibbs sampling to estimate the probability distribution. Formally, we let $z_{d}=\left\{ z_{d, 1}, z_{d, 2}, \ldots , z_{d, N_{d}}\right\}$ denote the treatment topics assigned according to the treatment day *d* and denote the treatment topic set by $\textbf{z}=\left\{ z_{d} \mid d \in \mathcal {D}\right\}$. This convention is also applied for treatment timestamps $\left\{ t_{d}, \textbf{t}\right\}$, and treatment activities $\left\{ a_{d}, \textbf{a}\right\}$. Specifically, for each treatment activity, we estimate the distribution of time stamp *t* and treatment topic *z* based on the following conditional probabilities $P(\varvec{z}, \varvec{t}, \varvec{a} \mid \alpha , \delta , \beta )$, which can be derived by marginalizing the joint probabilities in the following Eq. 4.

4 $$\begin{aligned} P(\textbf{z}, \varvec{t}, \varvec{a} \mid \alpha , \delta , \beta )=P(\varvec{z} \mid \alpha ) P(\varvec{t} \mid \textbf{z}, \delta ) P(\varvec{a} \mid \textbf{z}, \varvec{t}, \beta ) \end{aligned}$$

For $P(\textbf{z} \mid \alpha )$, which we can approximate by Gibbs sampling, is given by:

5 $$\begin{aligned} P(\textbf{z} \mid \alpha ) \propto \prod _{d=1}^{|\mathcal {D}|} \frac{\prod _{k=1}^{K} \Gamma \left( n_{d, k}+\alpha _{k}\right) }{\Gamma \left( \sum _{k=1}^{K} n_{d, k}+|K|\alpha \right) } \end{aligned}$$

where $\Gamma (\cdot )$denotes the gamma function and $n_{d, k}$ denotes the number of observed treatment days d assigned to treatment topic *k*.

For $P(\varvec{t} \mid \textbf{z}, \delta )$, which we can approximate by Gibbs sampling, is given by:

6 $$\begin{aligned} P(\varvec{t} \mid \textbf{z}, \delta ) \propto \prod _{k=1}^{K} \frac{\prod _{t=1}^{|T|} \Gamma \left( q_{k, t}+\delta _{t}\right) }{\Gamma \left( \sum _{t=1}^{|T|} q_{k, t}+|T|\delta \right) } \end{aligned}$$

where *T* denotes the universe of treatment days, $q_{k, t}$ denotes the number of times the observed timestamp *t* is assigned to treatment topic *k*.

For $P(\varvec{a} \mid \varvec{z}, \varvec{t}, \beta )$, which we can approximate by Gibbs sampling, is given by:

7 $$\begin{aligned} P(\varvec{a} \mid \textbf{z}, \varvec{t}, \beta ) \propto \prod _{k=1}^{K} \prod _{t=1}^{|T|} \frac{\prod _{a=1}^{|A|} \Gamma \left( m_{k, t, a}+\beta _{a}\right) }{\Gamma \left( \sum _{a=1}^{|A|} m_{k, t, a}+|A|\beta \right) } \end{aligned}$$

where *A* denotes the universe of treatment activities, and $m_{k, t, a}$ denotes the number of times the observed treatment activity *a* is assigned to treatment topic *k* with timestamp *t*.

The goal of our model is to derive the Gibbs sampling approximation distribution $P\left( z_{d, i}=k \mid z_{d,-i}, \varvec{t}, \varvec{a}, \alpha , \delta , \beta \right)$, where $Z_{d,-i}$ denotes the treatment topics except the current treatment topic. According to the above sampling process, the approximate probability distribution can be derived as follows.

8 $$\begin{aligned} P\left( z_{d, i}=k \mid z_{d,-i}, \varvec{t}, \varvec{a}, \alpha , \delta , \beta \right) \propto{} & {} \frac{n_{d, k}+\alpha }{\sum _{k=1}^{K} n_{d, k}+|K|\alpha } \nonumber \\{} & {} \times \frac{q_{k, t}+\delta }{\sum _{t=1}^{|T|} q_{k, t}+|T|\delta } \nonumber \\{} & {} \times \frac{m_{k, t, a}+\beta }{\sum _{a=1}^{|A|} m_{k, t, a}+|A|\beta } \end{aligned}$$

Based on the above equation, the probability can be calculated that the current treatment activity *a* in treatment day d belongs to a specific treatment topic. In addition, it is possible to calculate the treatment timestamp corresponding to the treatment topic which the current treatment activity is assigned by the above equation. Thus, the three distribution probabilities are as follows.

9 $$\begin{aligned} \theta _{d, k}=\frac{n_{d, k}+\alpha }{\sum _{k=1}^{K} n_{d, k}+|K|\alpha } \end{aligned}$$

10 $$\begin{aligned} \varphi _{k, t}=\frac{q_{k, t}+\delta }{\sum _{t=1}^{|T|} q_{k, t}+|T|\delta } \end{aligned}$$

11 $$\begin{aligned} \phi _{k, t, a}=\frac{m_{k, t, a}+\beta }{\sum _{a=1}^{|A|} m_{k, t, a}+|A|\beta } \end{aligned}$$

Details of the derivation are in Appendix A. We summarize the whole algorithm flow as Algorithm 1 shown.

**Figure:**
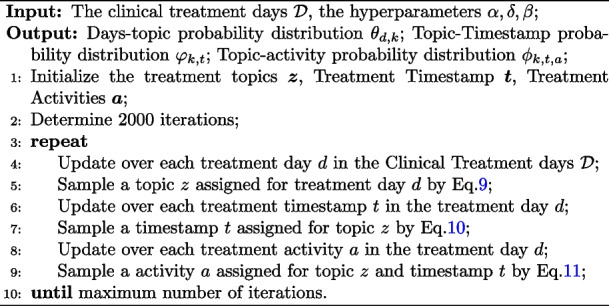
**Algorithm 1** The Proposed TTM Algorithm

## Results

In this section, we conducted extensive experiments to answer the following research questions.

**RQ1**: What about the effectiveness of our designed framework? Can it provide better performance compared to classical and state-of-the-art approaches?

**RQ2**: What about the interpretability of our model?

**RQ3**: What is the final clinical pathway model?

To address the above questions, this section evaluates the effectiveness of the proposed method. First, we present the dataset we used, the baseline method, the evaluation metrics, and the configuration of our method. In addition, we give the performance comparison of our method with classical and state-of-the-art methods. Finally, We visualized the final results.

### Experimental settings

#### Dataset description

The dataset used in this paper was extracted from the EMRs database of a first-rate hospital, which is among top 20 university hospitals in China. In the experiment, we extract the specific care procedure records of breast cancer patients from the EMR database. Additionally, we have removed undisclosed and incomplete medical records in our data, such as patient deaths or transfers during treatment. The hospitalization records kept are shown in Table 2, including disease name, treatment category, number of traces, number of activities, maximum length of stay (Max LOS), minimum length of stay (Min LOS), and average length of stay (Avg LOS). From the data, it was found that some patients are discharged with a very short hospital stay, but others require an exceptionally long stay to be discharged, which is reflecting the diversity of different treatment patterns in the specific care of breast cancer. Moreover, the data and experimental methods do not involve any sensitive and private information of the medical records, which has already been removed at the data pre-processing stage.

**Table 2:** Satistics of our dataset

Class	Trace	Activity	Activity type	Avg LOS	Min LOS	Max LOS
Radiotherapy	450	101883	237	13.05	2	85
Surgery	500	41706	259	11.74	2	169
Chemotherapy	500	27402	334	7.66	2	65

**Fig. 4:**
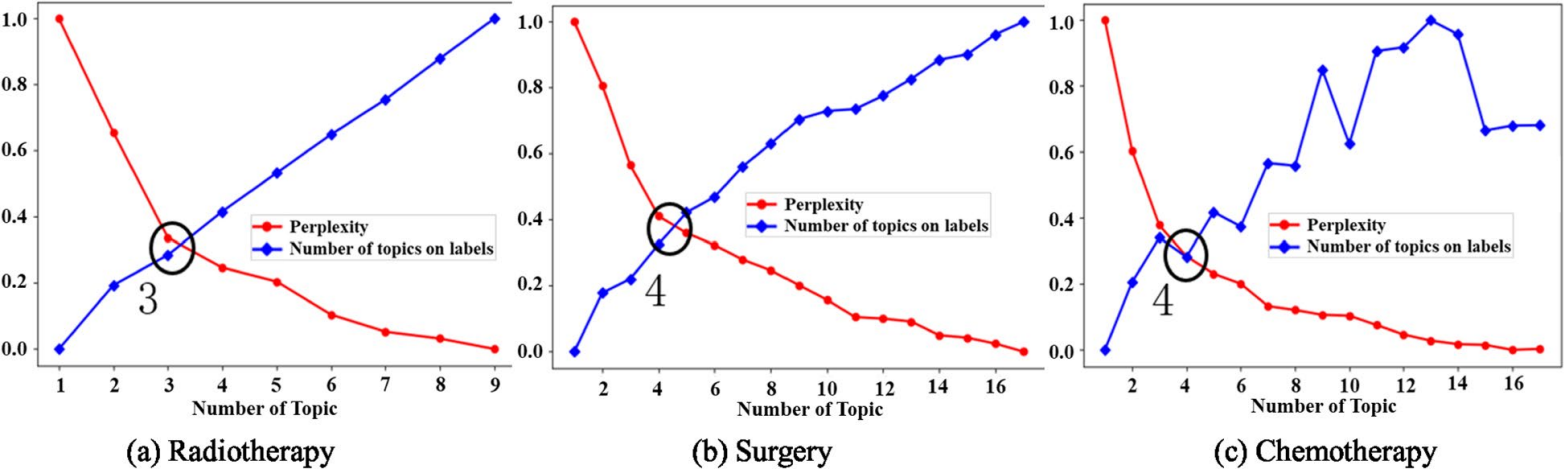
The topic number selection strategy

**Table 3:** The common set of treatment activities contained in the breast cancer treatment processes

Abbreviation	Description	Abbreviation	Description	Abbreviation	Description
$A_{0}$	Blood Glucose Test	$A_{16}$	General Anesthesia	$A_{32}$	Aminotrimadol Tablets
$A_{1}$	ECG	$A_{17}$	Removal of stitches	$A_{33}$	Granisetron Capsules
$A_{2}$	Blood Coagulation	$A_{18}$	Radical Surgery	$A_{34}$	Tamoxifen Citrate
$A_{3}$	Color Ultrasound	$A_{19}$	Pre-operative chest strap	$A_{35}$	Ubenimex Tablets
$A_{4}$	Blood Routine Examination	$A_{20}$	First-Grade Nursing	$A_{36}$	Fixed Irradiation
$A_{5}$	Liver Function	$A_{21}$	Second-Grade Nursing		
$A_{6}$	Kidney Function	$A_{22}$	Third-Grade Nursing		
$A_{7}$	CA15-3	$A_{23}$	Water Fasting		
$A_{8}$	Blood Fat Test	$A_{24}$	Capecitabine Tablets		
$A_{9}$	CT Scan	$A_{25}$	Letrozole Tablets		
$A_{10}$	MR Enhanced	$A_{26}$	Tropisetron Hydrochloride		
$A_{11}$	Thymopentin	$A_{27}$	Pantoprazole Sodium		
$A_{12}$	Sodium Chloride Injection	$A_{28}$	Levocarnitine Injection		
$A_{13}$	Glucose Injection	$A_{29}$	Cyclophosphamide Injection		
$A_{14}$	Pre-operative Skin Preparation	$A_{30}$	Sodium Deoxynucleotide Injection		
$A_{15}$	Drainage Measurement	$A_{31}$	Zoledronic Acid Injection		

#### Evaluation measurement

The model leverages Gibbs sampling to derive topic distributions $\theta _{d, k}$ , $\varphi _{k, t}$ and $\phi _{k, t, a}$. The treatment topics and corresponding treatment timestamps for each treatment day can be inferred from the topic distributions. We asked hospital doctors to assess the quality of the discovery topics by judging the corresponding relationships between treatment topics and treatment activities.

- **Treatment topic coherence:** Based on previous work [11], we adopt Top-k treatment activity to evaluate the consistency of the topic model, i.e., different topics contained Top-k treatment activities. We selected TOP-10 activities from each topic to calculate the topic coherence.
- **Treatment topic interpretability:** Taking into account the evaluation metrics of the study [6], we still adapt *NKQM*@*N*, an expanded form of NGCD [23] evaluation metrics, as our metrics to evaluate the ranking results. We asked three doctors to mark the top 20 terms for each topic as very relevant (score 2), relevant (score 1), or not relevant (score 0). The final score was determined by a voting strategy among the three doctors.
  12 $$\begin{aligned} N K Q M @ N=\frac{1}{K} \sum \limits _{k=1}^{K} \frac{\sum _{j=1}^{N} \frac{\textrm{score}\left( M_{k, j}\right) }{\textrm{log} (j+1)}}{Z_{N}} \end{aligned}$$
- ** Visualization Of clinical pathway model:** We statistically visualized the tuple consisting of the treatment topics and the corresponding time-stamped according to the average length of stay of the patients. On each treatment day, the probability distribution of each topic is visualized.

#### Baselines

We compare our model with the following baseline methods, including some classical methods.

- **Kmeans**. Kmeans (one of the most popular clustering algorithms) [24] with TF-IDF weights as our comparison, where TF-IDF is the product of words’ frequencies and inverse document frequencies. We treated every patient daily activity as a document for Kmeans and calculated TF-IDF for every activity.
- **Hierarchical Clustering**. Hierarchical clustering [25] attempts to divide the dataset at different levels, thereby forming a tree-like clustering structure. The dataset can be partitioned either by a “bottom-up” aggregation strategy or by a “top-down” splitting strategy.
- **Traditional LDA**.We apply the LDA model used in the study [6] as our comparison method. It considers the treatment days of the treatment processes as documents and the treatment activities as words, thereby discovering the treatment topics for each treatment day.

#### Topic number selection

An appropriate number of topics *K* is critical to the performance of LDA. In general, there are two main approaches to determine the number of topics *K*, human-defined and perplexity-based. In this study, we calculate the perplexity scores of the EMRs for specific diseases to determine the number of latent treatment topics. In information theory, perplexity is a measure of how well a probability distribution model predicts a sample. A model with a low perplexity probability distribution is better at predicting a sample. As shown in [21], the perplexity decreases as the number of topics *K* increases monotonically in the test data, and a lower perplexity score indicates better generalization performance of the model. In contrast, an excessive number of topics can additionally increase the complexity of the model. Therefore, in general, we take the *K* value corresponding to the first inflection point of the perplexity change curve as the optimal number of topics. For the remaining parameter settings, we keep them consistent with the research [6] ($\alpha =1.0, \beta =0.01$ and iterations = 2000).

13 $$\begin{aligned} \text {Perplexity }=\exp \left[ -\frac{\sum _{a \in \mathcal {D}} \textrm{log} p(a \mid \mathcal {D})}{\sum _{a \in \mathcal {D}}|a|}\right] \end{aligned}$$

where $|a|$ denotes the number of clinical activities in *a* and $\mathcal {D}$ denotes all treatment days for the particular disease. As shown in Fig. 4, for breast cancer radiation treatment, we choose the intersection $\textrm{K} \approx 4$ as radiation topic number, and this setting is also applied for surgery topic number to $\textrm{K} \approx 4$ and chemotherapy topic number to $\textrm{K} \approx 3$.

### Topic coherence (RQ1)

In this section, we first qualitatively evaluate the superiority of our method in terms of topic extraction methods. A partial set of abbreviations for breast cancer treatment activities is given in Table 3, where $A_{0} \sim A_{10}$ belong to examinations, $A_{11} \sim A_{19}$ to surgical operations, $A_{20} \sim A_{22}$ to nursing care, and $A_{23} \sim A_{36}$ to drugs administration. According to the abbreviation table of treatment activities in Table 3, Table 4 gives the Top-10 treatment activities discovered by each method under different topic labels. For each treatment topic, we select the top activity according to the topic-treatment activity distribution $\phi$ as the representation of the topic, and then invited doctors to label each topic based on the top activities.

**Table 4:** Comparisons with different methods on different topic labels performance, where the topic labels* are determined by the doctors based on the probability distribution of the corresponding topic

	

For LDA and MMT, we rank the activities of each topic by the probability distribution. For Kmeans and hierarchical cluster, we rank the activities of each cluster by the Euclidean Distance (ED) between activities

For the topic model, coherence measures the consistency of the top words in each topic, which is important for the interpretability of the topic. In the medical scenario, we focus on whether the top activities in each topic are all centered on the same treatment topic. Based on the results in Table 4, we can summarize the following conclusions, Where gray marks indicate treatment activities that do not belong to the treatment topic.

First, for radiotherapy treatment patients with breast cancer, we select the treatment topic of admission examination for method comparison. From the results, it can be observed that Kmeans and hierarchical clustering are generally effective, with 20-30% of the treatment activities discovered not belonging to this topic label. For the traditional LDA method, there is a 10% probability of discovering treatment activities that do not belong to that topic label and mainly belong to nursing topic. In comparison, our method performs significantly better than several other baseline methods.

Secondly, for surgery treatment patients with breast cancer, we select the topic label of surgical operation for the comparison of methods. From the results in the table, it can be observed that the comparison methods are significantly worse than our method, because our method has fewer treatment activities that do not belong to the topic label than the other baseline methods. However, our method can also be wrong if the treatment activity is used very frequently during the treatment.

Finally, for breast cancer chemotherapy patients, our method also shows better performance. While other baseline methods all present treatment activities that do not belong to the topic label, our method does not present other types of treatment activities under the TOP-10 of that topic label. In particular, for the LDA mistake performance, our method avoids this situation effectively by adopting the treatment time stamp.

### Topic interpretability (RQ2)

To prove the topic interpretability of our model, We adopt *NKQM*@*N* as our evaluation metric. We consider the scores determined by hospital doctors to yield comparative results of each method. As shown in Table 5, it is observed that TTM is remarkably better than other baseline methods across various *N* of *NKQM*.

**Table 5:** Comparisons with different methods on *NKQM*@*N* performance

Methods	Radiotherapy			Surgery			Chemotherapy		
	$N=5$	$N=10$	$N=20$	$N=5$	$N=10$	$N=20$	$N=5$	$N=10$	$N=20$
Kmeans	0.7047	0.6934	0.6538	0.6624	0.6875	0.6467	0.6855	0.6726	0.6328
Hier-Cluster	0.8026	0.7752	0.7628	0.7843	0.7652	0.7587	0.8128	0.7945	0.7831
LDA	0.8467	0.8159	0.7994	**0.8363**	0.8244	0.8098	0.8656	0.8478	0.8321
MMT	**0.8521**	**0.8320**	**0.8254**	0.8297	**0.8256**	**0.8186**	**0.8784**	**0.8542**	**0.8434**

Firstly, compared with traditional unsupervised learning methods, such as Kmeans, hierarchical clustering, it can be observed that our method exhibits significant advantages. Notably, in comparison with the traditional LDA method, our method adopts timestamps of treatment topics constraining the discovery of different treatment activities, prompting the consistency of topics and treatment activities. Although in some cases, such as surgery treatment, i.e., $N=5$, the traditional LDA method outperforms our method, in the majority of cases, our method is still significantly better than the traditional LDA. According to the topic interpretability analysis, the experimental results prove that our method is recognized by hospital experts and has certain reference value in the clinical application.

### Visualization of clinical pathway model (RQ3)

**Fig. 5:**
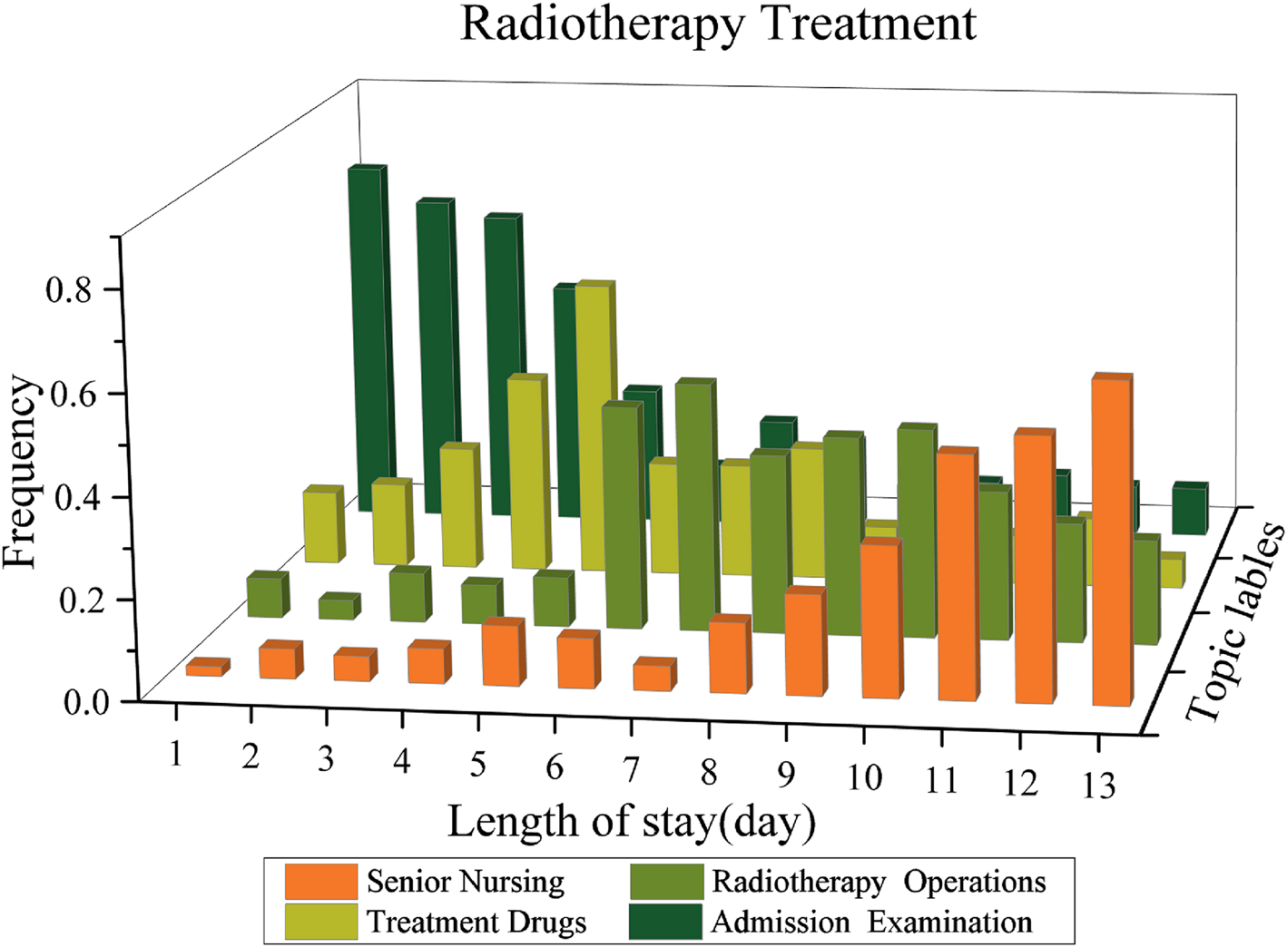
The discovered CP indicates Radiotherapy Treatment for patients with Breast cancer

**Fig. 6:**
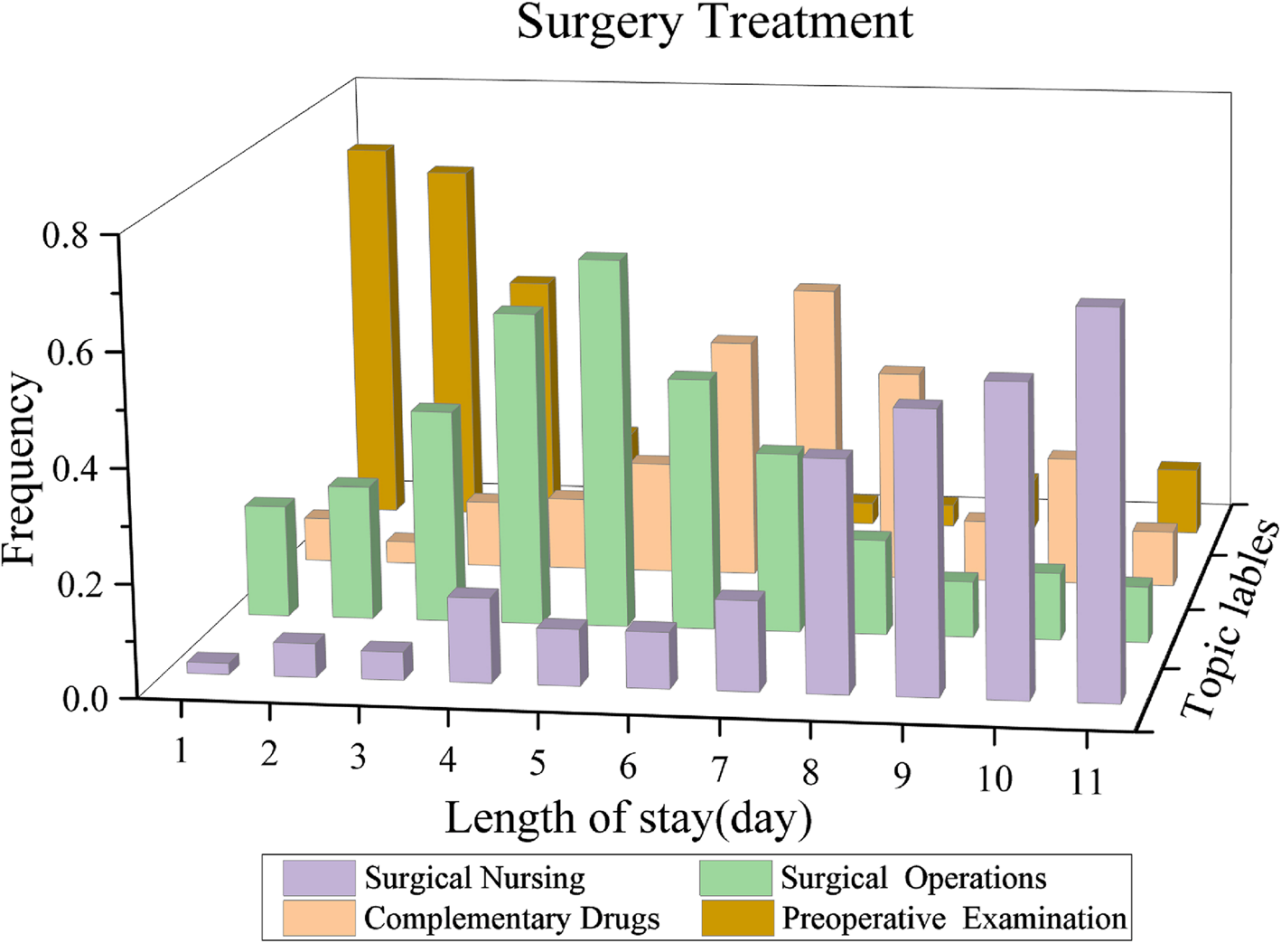
The discovered CP indicates Surgery Treatment for patients with Breast cancer

**Fig. 7:**
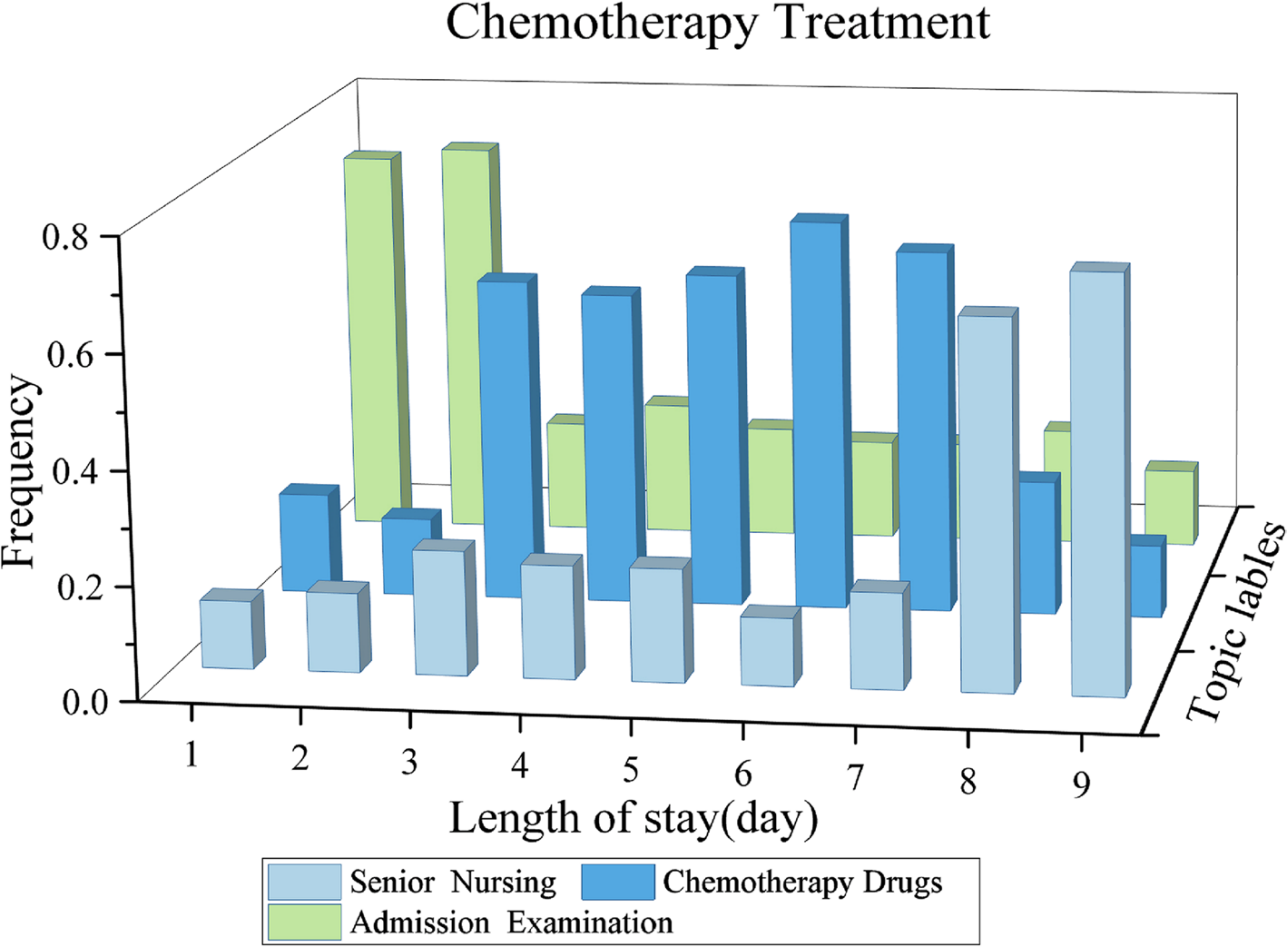
The discovered CP indicates Chemotherapy Treatment for patients with Breast cancer

After clustering the treatment activities by the TTM model, we can derive the CPs model $\varvec{C}$. We can visualize the CPs model $\varvec{C}$ based on patients’ length of stay (day). Figures 5, 6 and 7 show the mining results for each of the three treatment options for breast cancer.

The three treatment options are focused on the corresponding radiotherapy, surgery, and chemotherapy drugs topics, respectively. The rest of the treatment topics belong to the corresponding complementary treatment, which is also basically consistent with the national standard clinical pathway for breast cancer treatment.

From the illustrations of the discovered CPs, there are three main observations as follows.

- For the radiotherapy treatment, it can be seen that patients are required to perform admission examinations for the first five treatment days, which are determined by the doctor depending on the patient’s condition. The follow-up treatment focuses on radiology operations and the corresponding drugs. After completion of the radiotherapy treatment, patients will need to stay in hospital for nursing and observation.
- For the surgery treatment, patients need to complete pre-operative examinations within the first two treatment days of admission, this treatment process is essential for the surgery. The subsequent treatment focused on surgery and drugs. Before the patient can be discharged, the patient will need to stay in hospital for at least four or five days for post-operative care and recovery.
- For the chemotherapy treatment, patients will also need to be examined in hospital to determine whether they are medically suitable for chemotherapy. Once physically eligible, patients will receive chemotherapy treatment, which will last for one treatment period. Finally, the patient will need medical nursing for two days before discharge.

## Discussion

The experimental study analyzes the relationship between CPs and treatment activity with probabilistic generation model. We compared the proposed TTM model with the baselines. The experimental results demonstrate the validity of our TTM from three RQs. The specific explanations are presented as follows.

First, this study enriches the research on clinical pathway mining from treatment data. Compared with traditional clustering methods such as Kmeans and hierarchical clustering, our method adopts a probabilistic generative model to group and classify various treatment activities, which can better fit the characteristics of activity distribution in treatment data. Compared with the traditional topic model LDA, our method captures the temporal information in the treatment data, avoids the repetitive analysis of process mining, and ensures the temporal and generalized results of clinical pathway mining. Therefore, the topic results of the proposed method in RQ1 are more accurate than the baselines.

Second, the discovered CPs reveal key topics from the treatment data, forming backbones of CPs. This provides useful summaries of the treatment process , thus serving directly and explicitly as background knowledge for the targets of further analysis. As mentioned in RQ2, the discovered CPs are more consistent with the real treatment situation of patients in the care process, thus providing a higher subjective evaluation by experts than traditional methods.

Finally, from the perspective of mining path results in RQ3, our proposed method TTM provides a “data-to-model” approach to CPs redesign, which may be complementary to the prescribed expert knowledge-based approach. As a direct result, our method can be very useful for reducing the risk of complex and expensive CPs redesign projects.

Note that the current method has several limitations. First, in this study, only a portion of the treatment data was used to detect latent CPs. In clinical practice, many treatment decisions are made based on the patient’s physical and specific examination results [26], which is clearly beyond the data support of this study. Second, our model was only trained on the limited breast cancer dataset and has the possibility of overfitting.

## Conclusion

In this paper, we study the problem of CPs mining from EMRs in the medical field and provide a new method based traditional topic model. The method first adds the treatment day time message as the treatment timestamp to the topic model, which is solved iteratively by Gibbs sampling; based on the derived results, the EMRs data are converted into topic sequences, and the final clinical pathway model is obtained by statistical visualization. The topic model algorithm can well meet the needs of CPs for generalization and temporality.

Medical scenario is one of the important components of practical application scenarios, and our research provides new light on intelligent assisted medicine. Further, our research will focus on CPs discovery based on deep learning, CPs recommendation.

### Supplementary Information

**Additional file 1.**

## Data Availability

The data used in this paper are from the real data of the hospitals’ EMRs, and so it cannot be made freely available. Requests for access to these data should be made to the corresponding author.

## References

[1] Aspland E, Gartner D, Harper P (2021). Clinical pathway modelling: a literature review. Health Syst..

[2] Yang WS, Hwang SY (2006). A process-mining framework for the detection of healthcare fraud and abuse. Expert Syst Appl..

[3] dos Santos Garcia C, Meincheim A, Junior ERF, Dallagassa MR, Sato DMV, Carvalho DR (2019). Process mining techniques and applications-A systematic mapping study. Expert Syst Appl..

[4] Pika A, Wynn MT, Budiono S, Ter Hofstede AH, van der Aalst WM, Reijers HA (2020). Privacy-preserving process mining in healthcare. Int J Environ Res Public Health..

[5] Rebuge Á, Ferreira DR (2012). Business process analysis in healthcare environments: a methodology based on process mining. Inf Syst..

[6] Xu X, Jin T, Wei Z, Lv C, Wang J, TCPM: topic-based clinical pathway mining. In: 2016 IEEE first international conference on connected health: applications, systems and engineering technologies (CHASE). IEEE; 2016. p. 292–301.

[7] Huang Z, Lu X, Duan H (2013). Latent treatment pattern discovery for clinical processes. J Med Syst..

[8] Huang Z, Dong W, Ji L, Gan C, Lu X, Duan H (2014). Discovery of clinical pathway patterns from event logs using probabilistic topic models. J Biomed Inform..

[9] Huang Z, Dong W, Bath P, Ji L, Duan H (2015). On mining latent treatment patterns from electronic medical records. Data Min Knowl Disc..

[10] Huang Z, Dong W, Ji L, He C, Duan H (2016). Incorporating comorbidities into latent treatment pattern mining for clinical pathways. J Biomed Inform..

[11] Xu X, Jin T, Wei Z, Wang J. Incorporating topic assignment constraint and topic correlation limitation into clinical goal discovering for clinical pathway mining. J Healthc Eng. 2017(2017):1–13. 10.1155/2017/5208072 PMC5474282 29065617

[12] Jelodar H, Wang Y, Yuan C, Feng X, Jiang X, Li Y (2019). Latent Dirichlet allocation (LDA) and topic modeling: models, applications, a survey. Multimed Tools Appl..

[13] Munoz-Gama J, Martin N, Fernandez-Llatas C, Johnson OA, Sepúlveda M, Helm E (2022). Process mining for healthcare: characteristics and challenges. J Biomed Inform..

[14] Dallagassa MR, dos Santos Garcia C, Scalabrin EE, Ioshii SO, Carvalho DR. Opportunities and challenges for applying process mining in healthcare: A systematic mapping study. J Ambient Intell Humanized Comput. 2021(4):1–18.

[15] Diba K, Batoulis K, Weidlich M, Weske M (2020). Extraction, correlation, and abstraction of event data for process mining. Wiley Interdiscip Rev Data Min Knowl Disc..

[16] Mans R, Schonenberg H, Leonardi G, Panzarasa S, Cavallini A, Quaglini S, et al. Process mining techniques: an application to stroke care. Stud Health Technol Inform. 2008;136:573–78. 18487792

[17] Huang Z, Lu X, Duan H, Fan W (2013). Summarizing clinical pathways from event logs. J Biomed Inform..

[18] Neira RAQ, Hompes BFA, de Vries JGJ, Mazza BF, de Almeida SLS, Stretton E, et al. Analysis and optimization of a sepsis clinical pathway using process mining. In: International Conference on Business Process Management. Springer; 2019. p. 459–470.

[19] Kempa-Liehr AW, Lin CYC, Britten R, Armstrong D, Wallace J, Mordaunt D (2020). Healthcare pathway discovery and probabilistic machine learning. Int J Med Inform..

[20] Chen Y, Ghosh J, Bejan CA, Gunter CA, Gupta S, Kho A (2015). Building bridges across electronic health record systems through inferred phenotypic topics. J Biomed Inform..

[21] Blei DM, Ng AY, Jordan MI. Latent dirichlet allocation. J Mach Learn Res. 2003;3(Jan):993–1022.

[22] Newman D, Asuncion A, Smyth P, Welling M. Distributed algorithms for topic models. J Mach Learn Res. 2009;10(8).

[23] Shi N, Yu L, Sun L, Wang L, Lin C, Zhang R (2021). Deep heterogeneous network for temporal set prediction. Knowl-Based Syst..

[24] MacQueen J. Classification and analysis of multivariate observations. Proceedings of the 5th Berkeley Symposium on Mathematical Statistics and Probability, 1967. p. 281–297.

[25] Johnson SC (1967). Hierarchical clustering schemes. Psychometrika..

[26] Kaymak U, Mans R, Van de Steeg T, Dierks M, On process mining in health care. In: 2012 IEEE international conference on Systems, Man, and Cybernetics (SMC). IEEE; 2012. p. 1859–64.

