# Supplementary material for: Temporal topic model for clinical pathway mining from electronic medical records
Source: BMC Med Inform Decis Mak. 2024 Jan 23;24:20. doi: 10.1186/s12911-024-02418-1 (PMC10804581; doi:10.1186/s12911-024-02418-1)
Supplement: Supplementary file 1 — Additional file 1. [file 12911_2024_2418_MOESM1_ESM.docx]

Appendix A :The derivation process:$\begin{aligned} &P(\mathbf{z},\boldsymbol{t},\boldsymbol{a}\mid\alpha,\delta,\beta)=P(\mathbf{z}\mid\alpha)P(\boldsymbol{t}\mid\mathbf{z},\delta)P(\boldsymbol{a}\mid\mathbf{z},\boldsymbol{t},\beta) \\ &=\int P(\mathbf{z}\mid\boldsymbol{\theta})P(\theta\mid\alpha)d\boldsymbol{\theta}\int P(t\mid\mathbf{z},\Psi)\boldsymbol{P}(\Psi\mid\delta)d\Psi\int P(\boldsymbol{a}\mid\mathbf{z},t,\Phi)\boldsymbol{P}(\Phi\mid\delta)d\Phi\\ &=\int\prod_{d=1}^{|D|} \left( \prod_{i=1}^{N_{d}} P\left( z_{i}\mid\theta_{d} \right)P\left( \theta_{d}\mid\alpha\right) \right)d\Theta\\ &\int\prod_{d=1}^{|D|} \prod_{i=1}^{N_{d}} P\left( t_{d,i}\mid\varphi_{k,i} \right)\prod_{k=1}^{K} P\left( \varphi_{k,i}\mid\delta\right)d\Psi\\ &\int\prod_{d=1}^{|D|} \prod_{i=1}^{N_{d}} P\left( a_{d,i}\mid\phi_{k,t} \right)\prod_{k=1}^{K} \prod_{t=1}^{|T|} P\left( \phi_{k,t}\mid\beta\right)d\Phi\\ &=\int\prod_{d=1}^{|D|} \prod_{z=1}^{K} \theta_{d,k}^{n_{d,k}}\prod_{i=1}^{N_{d}} \left( \frac{\Gamma\left( \sum_{k=1}^{K} \alpha_{k} \right)}{\prod_{k=1}^{K} \Gamma\left( \alpha_{k} \right)}\prod_{z=1}^{K} \theta_{d,k}^{\alpha_{k}-1} \right)d\Theta\\ &\int\prod_{k=1}^{K} \prod_{t=1}^{|T|} \varphi_{k,t}^{q_{k,t}}\prod_{z=1}^{K} \left( \frac{\Gamma\left( \sum_{t=1}^{|T|} \delta_{t} \right)}{\prod_{t=1}^{|T|} \Gamma\left( \delta_{t} \right)}\prod_{t=1}^{|T|} \varphi_{k,t}^{\delta_{t}-1} \right)d\Psi\\ &\int\prod_{z=1}^{K} \prod_{t=1}^{|T|} \left( \prod_{a=1}^{|A|} \phi_{k,t,a}^{m_{k},ta}\frac{\Gamma\left( \sum_{a=1}^{|A|} \beta_{a} \right)}{\prod_{a=1}^{|A|} \Gamma\left( \beta_{a} \right)}\prod_{a=1}^{|A|} \phi_{z,t,a}^{\beta_{a}-1} \right)d\Phi\\ &\propto\prod_{d=1}^{\mathcal{|D|}} \frac{\prod_{k=1}^{K} \Gamma\left( n_{d,k}+\alpha_{k} \right)}{\Gamma\left( \sum_{k=1}^{K} n_{d,k}+|K|\alpha\right)}\cdot\prod_{k=1}^{K} \frac{\prod_{t=1}^{|T|} \Gamma\left( q_{k,t}+\delta_{t} \right)}{\Gamma\left( \sum_{t=1}^{|T|} q_{k,t}+|T|\delta\right)} \\ &\cdot\prod_{k=1}^{K} \prod_{t=1}^{|T|} \frac{\prod_{a=1}^{|A|} \Gamma\left( m_{k,t,a}+\beta_{a} \right)}{\Gamma\left( \sum_{a=1}^{|A|} m_{k,t,a}+|A|\beta\right)} \\ & \end{aligned}$ (A1)

With the Gibbs sampling process described above, we can derive the final conditional probabilities.

$\begin{aligned} P\left( z_{d,i}=k\mid z_{d,-i},t,a,\alpha,\delta,\beta\right)&\propto\frac{P(z,t,a,\alpha,\delta,\beta)}{P\left( z_{d,-i},t,a,\alpha,\delta,\beta\right)} \\ &\propto\frac{n_{d,k}+\alpha}{\sum_{k=1}^{K} n_{d,k}+|K|\alpha} \\ &\times\frac{q_{k,t}+\delta}{\sum_{t=1}^{|T|} q_{k,t}+|T|\delta} \\ &\times\frac{m_{k,t,a}+\beta}{\sum_{a=1}^{|A|} m_{k,t,a}+|A|\beta} \end{aligned}$ (A2)
